# Design and Control of a Polycentric Knee Exoskeleton Using an Electro-Hydraulic Actuator

**DOI:** 10.3390/s20010211

**Published:** 2019-12-30

**Authors:** Taesik Lee, Dongyoung Lee, Buchun Song, Yoon Su Baek

**Affiliations:** Department of Mechanical Engineering, Yonsei University, Seoul 03722, Korea; lts9828@naver.com (T.L.); frh1024@naver.com (D.L.); theme7749@gmail.com (B.S.)

**Keywords:** polycentric structure, knee exoskeleton, pump-controlled electro-hydraulic actuator (EHA), sliding mode control (SMC)

## Abstract

An exoskeleton robot helps the wearer with mechanical forces by identifying the wearer’s intentions and requires high energy efficiency, sufficient load capacity, and a comfortable fit. However, since it is difficult to implement complex anatomical movements of the human body, most exoskeleton robots are designed simply, unlike the anatomy of real humans. This forces the wearer to accept the robot’s stiffness entirely, and to use energy inefficiently from the power source. In this paper, a simple 1 degree of freedom (DoF) structure, which was mainly used in the knees of exoskeleton robots, was designed with a polycentric (multi-axial) structure to minimize the misalignment between wearer and robot, so that torque transfer could be carried out efficiently. In addition, the overall robot system was constructed by using an electro-hydraulic actuator (EHA) to solve the problems of the energy inefficiency of conventional hydraulic actuators and the low load capacity of conventional electric actuators. After the configuration of the hardware system, the sliding mode controller was designed to address the EHA nonlinear models and the uncertainty of the plant design. This was configured as Simulink for the first verification, and the experiment was conducted by applying it to the actual model to demonstrate the performance of the sliding mode control. In this process, an optical rotary encoder was used as the main feedback sensor of the controller. The proposed polycentric knee exoskeleton robot system using the EHA was able to reach the desired target value well despite the presence of many model uncertainties.

## 1. Introduction

An exoskeleton system is a kind of wearable robot that analyzes the wearer’s intentions and assists the wearer with mechanical forces. The exoskeleton robot is currently being used in various fields, including industrial, military, medical, and rehabilitation [1,2], and related research is actively being carried out to suit the purpose of each field. In the past, robots that directly helped people, such as for human power augmentation, were mainly developed in industrial and military fields. However, in modern times robots are being developed in a form used to closely assist people in their daily lives or to help them with rehabilitation training. These changes are strongly related to the aging of society [3]. As the elderly population has grown, the number of patients with musculoskeletal or neurological disorders likely to cause gait difficulties has increased relatively [4]. Additionally, the lack of physiotherapists to train these patients is one of the reasons for the shift to robot-assisted rehabilitation. Conventional manual therapies rely mainly on the experience of therapists and require high-intensity repetitive training, which is difficult to achieve due to the lack of therapists compared to the number of patients [5]. Inevitably, not only the patients but also the rehabilitation medical community has sought rehabilitation aids to solve these problems, which has led to the development of the lower limb rehabilitation exoskeleton robot.

These lower limb robots reduce the burden of wearer fatigue by assisting with muscle strength, and they help with rehabilitation training so that patients can overcome physical disabilities and lead their daily lives like ordinary people. In fact, there have been cases where patients with cerebral palsy have improved their walking abilities by training themselves to walk on treadmills wearing exoskeleton robots [6]. Similar studies have demonstrated to some extent the effectiveness of exoskeleton robots, which has further raised people’s interest in the relevant fields.

Recently, it has been reported that the development of various mechanisms for exoskeleton robots, as well as the systematization of training methods and the enhancement of control methods, are being carried out together [7]. Typical examples of lower limb exoskeleton robots that have become commercialized include Lokomat (Hocoma, Volketswil, Switzerland), ReWalk Personal (ReWalk Robotics, Marlborough, MA, USA), and HAL (Cyberdyne, Tsukuba, Japan) [8,9,10]. Several rehabilitation exoskeleton robots specialized for knee joints have been introduced in the literature, which are in line with the focus of this study [11]; these include DGO/Lokomat [12,13], which has a ball screw driven by a DC motor; LOPES [14,15,16], which has a Bowden cable driven by series elastic actuators (SEAs); KNEXO [17,18], based on pleated pneumatic artificial muscles (PPAMs); and ALEX [19], which contains electrically operated linear actuators. The AlterG Bionic leg, made in the United States, is also an example of a commercialized knee joint exoskeleton robot [20].

**Figure 1:**
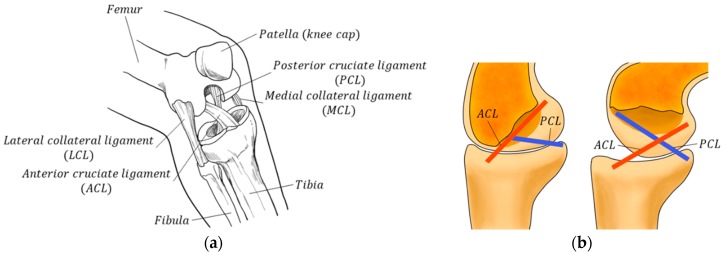
Overview of knee anatomy: (**a**) anatomical structure of the human knee; (**b**) changes in the shape and tension of the anterior cruciate ligament (ACL) and posterior cruciate ligament (PCL).

When looking at the knee structures presented earlier, most of the robots use a simply hinged 1 degree of freedom (DoF) system, which is designed to facilitate system control through the simplification of dynamic models; it also serves the purpose of improved durability. In reality, however, human knee joints do not simply rotate around one axis. From an anatomical point of view, there are ligaments in the knee, called the anterior cruciate ligament (ACL) and posterior cruciate ligament (PCL) as shown in Figure 1, that connect the femur and tibia, and their sophisticated movements result in both gliding and rocking motions as well as rotation motion [21,22]. This motion is similar to the movement of the well-known four-bar linkage system and is also called a polycentric (multi-axial) system. The polycentric knee structure has the effect of shortening the length of the legs in the swing phase when walking, thus preventing foot drop and making it possible to walk in a manner that appears natural. Therefore, it is widely used for prosthesis or orthosis, and the torque applied to the hip joint can be managed efficiently depending on the location of the instantaneous center of rotation (ICR) for the ground reaction force [23]. As a result, a simple 1 DoF knee structure system does not take into account this anatomical knowledge, causing misalignment between the apparatus and the person, making the wearer feel uncomfortable, and causing loss of energy from the power source.

From another point of view, these exoskeleton robots should be able to transmit enough power to the wearer repeatedly for that purpose. Generally, power is transmitted using high-powered hydraulic actuators, energy-efficient electric actuators, light-weight pneumatic artificial muscles, or series elastic actuators (SEAs) with small impedance characteristics, as mentioned above. Since each actuator has clear advantages and disadvantages, the actuator is selected according to the robot designer’s intention or purpose [24]. For example, conventional hydraulic actuators have high output, but they are bulky and have problems with low energy efficiency. In contrast, for electric actuators, the configuration is simple and energy-efficient, but the holding capacity is poor under high loads. Pneumatic actuators can exert greater force with light weights and have the advantage of being flexible due to the use of soft material; however, there is a large amount of noise and energy loss at the stage of air compression and valve control. As for SEA, it has little impedance and good back-drivability [25], but the stiffness value is inherently small, which limits control performance. In this study, the focus was on passive training [7,26], specifically intensive and repetitive training, conducted on patients with exoskeleton robots; thus, a system with correspondingly high output characteristics was needed. Therefore, we had to choose a hydraulic system, a system known for problems of huge bulk, low energy efficiency, and flow rate leakage. However, in order to overcome these weaknesses, a solution was considered that used a hydraulic system operated by electrical power to control the exoskeleton through an electro-hydraulic actuator (EHA). The pump-controlled EHA is a more flexible system than the valve-controlled conventional hydraulic pump system. The pump-controlled EHA is precise in its control method and has excellent strength in positioning, so it shows good characteristics in following target values. In addition, the hydraulic circuit structure is simpler than the conventional hydraulic system, so it is small in volume, requires little oil leakage management because it does not use valves, and has high energy efficiency [27]. Although many studies focus on valve control systems, which have rapid rates of response [28,29], these systems, while responding at high speeds, require a constant pressure supply and accumulate many uncertainties by causing gradual leakage from valves. Thus, they are somewhat less energy-efficient and more difficult in terms of ensuring control performance. The more advanced the system is, the more important the problem of energy efficiency and the maintenance of accurate control performance are because they are directly related to the stability of the exoskeleton robot. Therefore, more advanced forms of research are essential to increase energy efficiency using the pump-controlled EHA.

In summary, in this study, a polycentric knee structure with a rotary encoder sensor was designed to minimize the misalignment between the wearer and robot and to ensure efficient torque delivery in order to compensate for the shortcomings of the simple 1 DoF knee joint system used in most exoskeleton robots. In addition, the overall robot system was constructed using a pump-controlled EHA with high power and energy efficiency. Generally, hydraulic actuator (HA) systems have high power but have low energy efficiency and are bulky. Therefore, we thought that the EHA system, which includes both the high power characteristics of HA and the superior energy efficiency of the electrical actuating system, was suitable for application in this system. After the configuration of the entire system, a sliding mode controller (SMC) was designed to address the mechanical uncertainty of the polycentric knee exoskeleton and the nonlinearity of the EHA unit. Mechanical uncertainty is typical of the nonlinear movement of the polycentric knee structure, and modeling uncertainty is caused by the presence of a single-rod cylinder and changes in mass. Nonlinearity in EHA units is the main cause, for example, of leakage from the movement of fluids. This is why an SMC with a feedback sensor, a nonlinear controller that can solve the model’s uncertainty problem, was used.

The remainder of this paper is structured as follows: The methods for designing the mechanisms for polycentric joints and for estimating the actual knee angle through sensors in the entire exoskeleton robot system, including the polycentric joints designed, are described in Section 2. The mathematical theory of motors, pumps, and cylinders, the main components of the EHA system used as a power source, is described in Section 3. Based on this, the dynamic equation of the exoskeleton robot is organized to define the entire plant system for nonlinear control. The process of designing the SMC by applying Lyapunov theory is dealt with in Section 4. Verification through experimentation and results is covered in Section 5, and results are discussed in Section 6. Finally, the conclusions of this paper are presented in the last section.

## 2. Mechanism Design of the Polycentric Knee Joint

**Figure 2:**
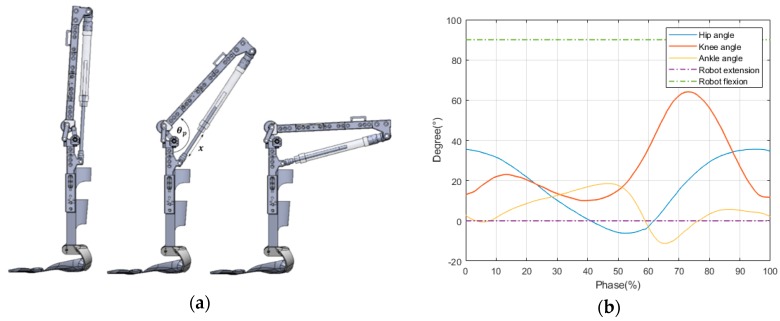
Range of motion of the designed polycentric knee exoskeleton: (**a**) design modeling of the polycentric knee exoskeleton; (**b**) normal angle and workspace of the robot during the gait cycle.

From an anatomical point of view, the ideal knee joint range of motion (ROM) is 0° to 135°, with the possibility of occasional hyperextension of 0° to 5°. However, the angular range of the knees used for walking is less, as shown in Figure 2b [30,31], which is why most prior studies limited the robot’s workspace to 0° to 90° [11]. Thus, in this study, as shown in Figure 2a, the robot’s knee movement angle was designed to be limited from 0° to 90°, meeting the range of biological knee motion required for walking.

Based on Gruebler’s equation [32,33], the number of degrees of freedom of the entire system was determined by the 1 DoF system, and the polycentric knee exoskeleton robot was designed to operate for the movement of one actuator. In addition, for polycentric joint structures, the links were constructed with the double-rocker inversion model meeting the Grashof condition [32,33,34], which limited the range of motion (ROM). This was to prevent impossible movements of the actual knee joint, such as hyperflexion or hyperextension.

**Figure 3:**
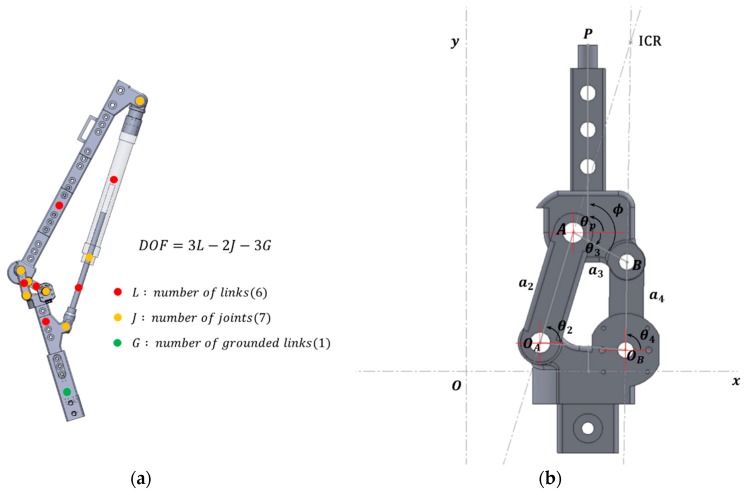
Design of the knee structure and determination of the number of degrees of freedom (DoF): (**a**) calculation point of the degrees of freedom; (**b**) configuration of a polycentric knee with coupler point P. ICR is instantaneous center of rotation.

Due to the complex structure of polycentric knee joints, as shown in Figure 3, there are many difficulties in directly observing the actual knee angle. Therefore, it is necessary to attach the observer AMT203-V rotary encoder (CUI Devices, United States) to a link in a polycentric structure to convert the observed values to the actual knee angle. The angle at which the rigidity contains any point P attached to the coupler for the ground can be calculated through the vector loop equation. This process means estimating the relative angles of the femur and tibia in the actual human body, which can be calculated using the following equations from earlier work [35]:

(1) $$\{\begin{array}{c} x_{B}=x_{OB}+a_{4}cos\theta_{4}=x_{OA}+a_{2}cos\theta_{2}+a_{3}cos\theta_{3}\\ y_{B}=y_{OB}+a_{4}sin\theta_{4}=y_{OA}+a_{2}sin\theta_{2}+a_{3}sin\theta_{3} \end{array}$$

$$a_{2}cos\theta_{2}=-a_{3}cos\theta_{3}+C_{1}$$

$$a_{2}sin\theta_{2}=-a_{3}sin\theta_{3}+C_{2}$$

where

$$\{\begin{array}{c} C_{1}=x_{OB}-x_{OA}+a_{4}cos\theta_{4}\\ C_{2}=y_{OB}-y_{OA}+a_{4}sin\theta_{4} \end{array}$$

(2) $$a_{2}{}^{2}=a_{3}{}^{2}+C_{1}{}^{2}+C_{2}{}^{2}-2a_{3}C_{1}cos\theta_{3}-2a_{3}C_{2}sin\theta_{3}.$$

In order to obtain information about the coupler link from $\theta_{4}$ measured by the encoder, the terms for $\theta_{2}$ must be removed. After removing the variables for an unobservable $\theta_{2}$ using the trigonometric formula, the $\theta_{3}$ can be determined by a generalized form of expression, such as Equation (3).

(3) $$Asin\theta_{3}+Bcos\theta_{3}=C$$

where

$$\{\begin{array}{c} A=-2C_{2}a_{3}\\ B=-2C_{1}a_{3}\\ C=a_{2}{}^{2}-a_{3}{}^{2}-C_{1}{}^{2}-C_{2}{}^{2} \end{array}$$

(4) $$sin\theta_{3}=\frac{2tan\left(\frac{\theta_{3}}{2}\right)}{1+{tan}^{2}\left(\frac{\theta_{3}}{2}\right)},cos\theta_{3}=\frac{1-{tan}^{2}\left(\frac{\theta_{3}}{2}\right)}{1+{tan}^{2}\left(\frac{\theta_{3}}{2}\right)}.$$

Equation (3) can be expressed as Equation (5) by substituting Equation (4), and Equation (5) is expressed as a quadratic equation for $tan\frac{\theta }{2}$, so the value of $\theta_{3}$ can be obtained using the quadratic formula.

(5) $$\left(B+C\right){tan}^{2}\frac{\theta_{3}}{2}-2Atan\frac{\theta_{3}}{2}-\left(B-C\right)=0$$

(6) $$\theta_{3}=2{tan}^{-1}\frac{A\pm \sqrt{A^{2}+B^{2}-C^{2}}}{B+C}$$

Finally, using this $\theta_{3}$, it is possible to determine the relative angle of any point P present in the coupler and the ground link using the equation expressed as follows:

(7) $$\theta_{p}=\theta_{3}+\phi .$$

**Figure 4:**
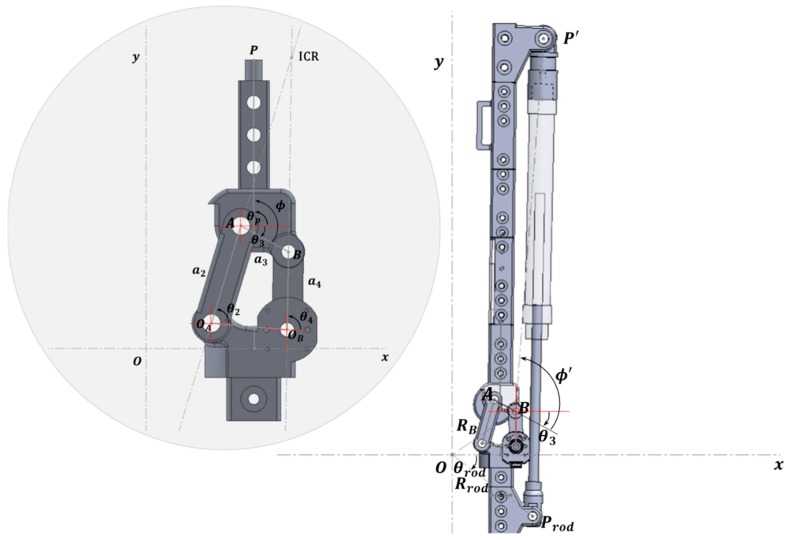
Decision of coupler point $P^{\prime }$ and $P_{rod}$ to calculate actuator length.

The polycentric knee exoskeleton robot controls the knee angle by changing the rod length of the cylinder using the EHA drive. Therefore, it is important to know the correlation between the two parameters, which can be inferred based on calculations within the absolute coordinate system as shown in Figure 4. The theoretical calculation is expressed as follows:

(8) $$R_{P^{\prime }}=R_{B}+R_{P^{\prime }B}$$

where

$$R_{P^{\prime }B}=\bar{P^{\prime }B}e^{j\left(\theta_{3}+\phi^{\prime }\right)}=\bar{P^{\prime }B}\left[\cos\left(\theta_{3}+\phi^{\prime }\right)+j\sin\left(\theta_{3}+\phi^{\prime }\right)\right].$$

In addition, the coordinates of the ground link within the absolute coordinate system can be expressed as follows:

(9) $$R_{rod}=\bar{P_{rod}O}e^{j\theta_{rod}}.$$

The relationship between $\theta_{p}$ and x can be determined by calculating the distance between the two points obtained from Equations (8) and (9). However, if calculations are made in this way, a process similar to Equations (1)–(7) must be repeated, which burdens the microcontroller unit (MCU) with excessive computations in processing the data. Thus, after considering that the above robot system was designed in 1 DoF, and that the two variables could be expressed in a bijective function, we converted the complex computation of trigonometric functions into a polynomial form to reduce computations within the MCU. To do this, the motion analysis function of Solidworks [36] was used to obtain the change in rod length (x) information regarding the changing angle ($\theta_{p}$).

**Figure 5:**
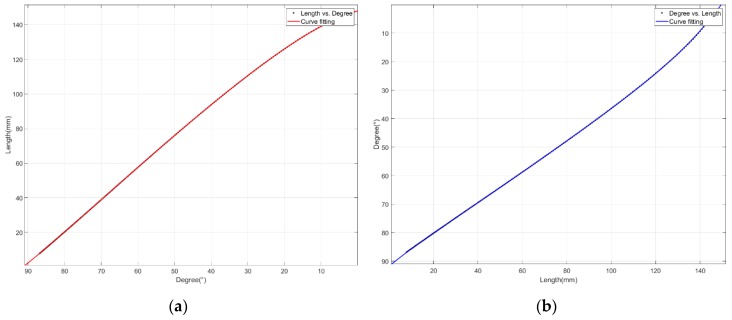
Polynomial curve fitting: (**a**) $\theta_{p}$ to x; (**b**) x to $\theta_{p}$.

Based on the data obtained from the simulation, as shown in Figure 5, curve fitting was performed and expressed in the fifth polynomial form. The relationship between $\theta_{p}$ and x is organized as shown in Equations (10) and (11), and the respective coefficients are listed in Table 1. The sum of square errors (SSEs) were 0.1781 and 1.241, and the root mean square errors (RMSEs) were 0.03 and 0.0792, respectively.

(10) $$x=p_{1}\theta_{p}{}^{5}+p_{2}\theta_{p}{}^{4}+p_{3}\theta_{p}{}^{3}+p_{4}\theta_{p}{}^{2}+p_{5}\theta_{p}+p_{6}$$

(11) $$\theta_{p}=q_{1}x^{5}+q_{2}x^{4}+q_{3}x^{3}+q_{4}x^{2}+q_{5}x+q_{6}$$

## 3. Mathematical Modeling of the Entire System, Including the EHA

Section 3 describes the mechanical theory of the axial-piston pump with brushless DC (BLDC) motor and single-rod cylinder, the components of the EHA system used as the power source for exoskeleton robots. It also introduces the process of finding the ideal plant equation for designing nonlinear controllers by establishing the dynamic equation of polycentric knee exoskeleton robots that include this system.

**Figure 6:**
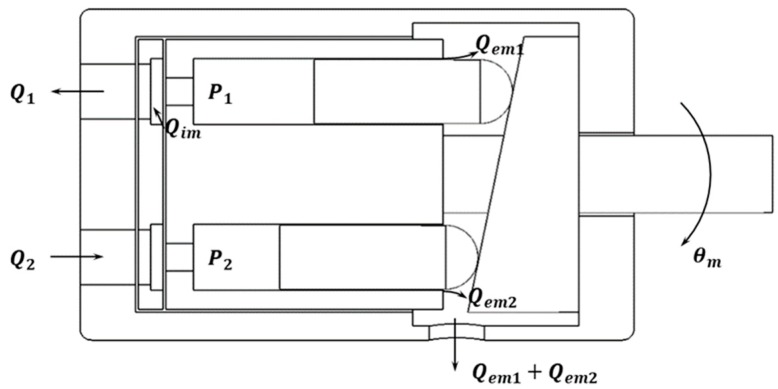
Schematic diagram of an axial-piston pump: cam plate; shaft; cylinder block and piston shoes; cylinder valve.

Figure 6 is the schematic diagram of the axial-piston pump, a system that converts the motor’s rotational force to flow rate. The pump transmits power from the motor through the shaft, which moves at a constant flow rate to the cylinder. The pump dynamics of the steady-state continuity equation can be written, as in an earlier study [37], as follows:

(12) $$\{\begin{array}{c} D_{m}{\dot{\theta }}_{m}+C_{im}\left(P_{2}-P_{1}\right)-C_{em}P_{1}-Q_{1}=0\\ Q_{2}-C_{em}P_{2}-C_{im}\left(P_{2}-P_{1}\right)-D_{m}{\dot{\theta }}_{m}=0 \end{array}$$

where $D_{m}$ is the ideal volumetric displacement of the motor-pump and ${\dot{\theta }}_{m}$ represents the motor shaft speed. The pump is connected by two lines, $Q_{1}$ and $Q_{2}$, in the chambers. $Q_{1}$ is the return flow from the motor, $Q_{2}$ is the forward flow to the motor, and $C_{im}$ and $C_{em}$ are the internal (or cross-port) and external leakage coefficients, respectively. $P_{1}$ and $P_{2}$ are the pressure in the return and forward chambers, respectively. After subtracting the two expressions of Equation (12), the flow rate variables can be summarized and expressed as Equation (13).

(13) $$Q_{L}=\frac{Q_{1}+Q_{2}}{2}=D_{m}{\dot{\theta }}_{m}-(C_{im}+\frac{C_{em}}{2})P_{L}$$

where

$$P_{L}=P_{1}-P_{2}.$$

$Q_{L}$ is the load flow, and $P_{L}$ is the pressure difference between $P_{1}$ and $P_{2}$.

**Figure 7:**
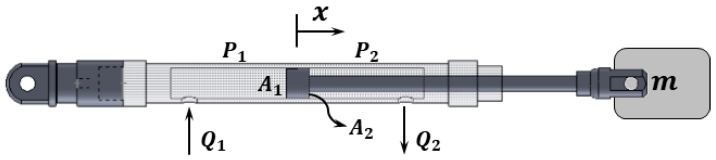
Structure of the hydraulic single-rod cylinder.

Figure 7 shows the structure of the single-rod cylinder, the part where the flow rate moved by the motor-pump system is actually operated as the actuator. Unlike a double-rod cylinder, the I/O length variation of the cylinder rod is not constant for the same flow input, as the two chambers have different internal areas and volumes. The mechanical formulas for the cylinders are as follows [38]:

(14) $$\{\begin{array}{c} \frac{V_{1}}{\beta_{e}}{\dot{P}}_{1}=-A_{1}\dot{x}-C_{im^{\prime }}\left(P_{1}-P_{2}\right)-C_{em1^{\prime }}\left(P_{1}-P_{r}\right)+Q_{1}\\ \frac{V_{2}}{\beta_{e}}{\dot{P}}_{2}=-A_{2}\dot{x}-C_{im^{\prime }}\left(P_{1}-P_{2}\right)-C_{em2^{\prime }}\left(P_{2}-P_{r}\right)-Q_{2} \end{array}$$

where

$$V_{1}=V_{1}^{0}+A_{1}x$$

$$V_{2}=V_{2}^{0}+A_{2}x.$$

$V_{1}^{0}$ and $V_{2}^{0}$ are the first and second chamber volumes of the initial condition, respectively, and $\beta_{e}$ is the bulk modulus of hydraulic oil. $C_{im^{\prime }}$ is the coefficient of the internal leakage in the cylinder. $C_{em1^{\prime }}$ and $C_{em2^{\prime }}$ are the coefficients of the external leakage in each respective chamber line. The $Q_{1}$ and $Q_{2}$ presented in Equation (12) are the supply flow and return flow in the cylinder, respectively. $A_{1}$ and $A_{2}$ represent the area of each respective chamber. Since it is complicated to observe all the variables in Equation (14), it is necessary to simplify and organize the two formulas to eliminate the number of variables to be observed. However, as mentioned above, double acting single-rod cylinders are difficult to define using one formula because of the different volumes and cross-sectional areas of the chambers inside. Therefore, assuming the mean condition $V_{t}=V_{1}+V_{2}$, $\bar{A}=\frac{1}{2}\left(A_{1}+A_{2}\right)$, at risk of introducing some error [39], the formula is reduced to derive an expression for the gradient of the following pressure difference:

(15) $${\dot{P}}_{L}=\frac{4\beta_{e}}{Vt}\left(Q_{L}-\bar{A}\dot{x}-C_{L}P_{L}\right).$$

The mechanical sub-model formula for explaining the motion of a 1 DoF cylinder, based on Newton’s second law and Pascal’s law, is as follows:

(16) $$m\ddot{x}=P_{L}\bar{A}-f\left(x\right)-F_{L}$$

where

$$f\left(x\right)=B\dot{x}+Kx.$$

B is the damping coefficient, and K is the spring coefficient. $F_{L}$ is a disturbance that causes modeling uncertainty. From Equation (16), the differential Equation (17) is derived, as shown below, by combing the axial-pump equation and the cylinder equation:

(17) $$m\dddot{x}=\frac{4\beta_{e}}{Vt}\left(Q_{L}-\bar{A}\dot{x}-C_{L}P_{L}\right)\bar{A}-\dot{f}\left(x\right)-{\dot{F}}_{L}$$

where

$$Q_{L}=D_{m}{\dot{\theta }}_{m}={\tilde{s_{r}}}^{-1}D_{m}u.$$

Here, u and $\tilde{s_{r}}$ denote the motor shaft speed and the scale factor to unify the unit. This u is operated by the control input on the controller described in Section 4.

## 4. Design of a Sliding Mode Control

**Figure 8:**
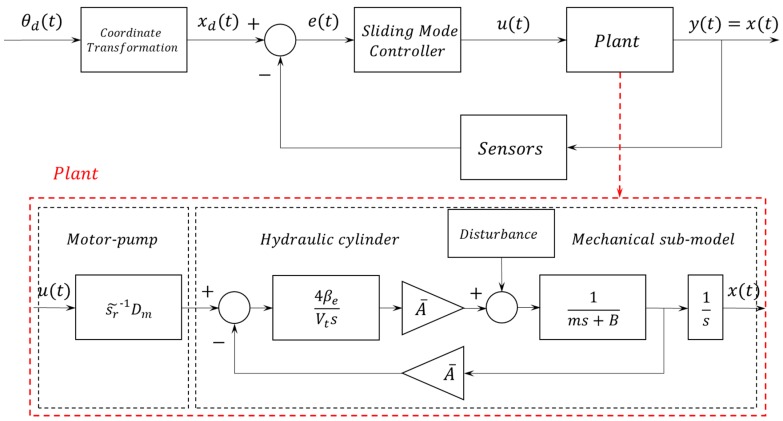
Sliding mode control scheme.

In general, the purpose of controller design is to regulate control inputs into plant systems to ensure that the output of the plant is well followed by the reference input. Figure 8 is a schematic of the sliding mode control and includes the contents covered in Section 2 and Section 3. Material related to the coordinate transformation was introduced in Section 2, and the induction process of the plant model was dealt with in Section 3. Between these blocks, it is the role of the sliding mode controller to control the amount of control input (u) with real-time information obtained through the feedback sensor.

A sliding mode control was developed in order to minimize the model uncertainties caused by nonlinearity of the EHA unit and an incomplete mechanical formula. The state equation for nonlinear systems can be expressed in the following generalized form:

(18) $$x^{\left(n\right)}=f\left(x\right)+b\left(x\right)u.$$

By defining a time-varying sliding surface S(t) in the state-space $R^{\left(n\right)}$ by a scalar equation s(x;t)=0 [40], the following is obtained:

(19) $$s\left(x;t\right)={\left(\frac{d}{dt}+\lambda \right)}^{n-1}\tilde{x}$$

where

$$\tilde{x}=x-x_{d}=e.$$

Here, λ is strictly a positive constant. In this system, a tertiary filter structure (n=3) consisting of a weighted sum of position error and velocity error is defined as follows:

(20) $$n=3:\;s\left(x;t\right)={\left(\frac{d}{dt}+\lambda \right)}^{2}\tilde{x}=\ddot{\tilde{x}}+2\lambda \dot{\tilde{x}}+\lambda^{2}\tilde{x}=\ddot{e}+2\lambda \dot{e}+\lambda^{2}e.$$

Once the dimension (n) of the sliding variable has been determined, the appropriate selection of the control input (u) to make it s=0 is carried out. The basic theory is based on the method of selecting the Lyapunov function and its contents, which are expressed as follows:

(21) $$V=\frac{1}{2}s^{2}$$

(22) $$\frac{dV}{dt}=s\dot{s}\leq -\eta \left|s\right|\left(\leq 0\right).$$

For the system to be stable, the condition of zero is met only when s=0, and the sliding condition is determined using Equation (22). The dynamic formula of the ideal plant can be presented as shown in Equation (23), but it is very difficult to know exactly what the actual plant information is, and therefore another estimation plant equation is defined.

(23) $$\{\begin{array}{cc} Plant & :\;\;\;\dddot{x}=f\left(x,\dot{x},\ddot{x}\right)+bu\\ Model & :\;\;\;\dddot{x}=\hat{f}\left(x,\dot{x},\ddot{x}\right)+\hat{b}u \end{array}$$

$$Assumption:\;\left|\hat{f}-f\right|\leq F\left(x,\dot{x}\right)$$

$$0<b_{min}\leq b\leq b_{max}\to \hat{b}=\sqrt{b_{min}b_{max}}$$

$$\beta^{-1}\leq \frac{\hat{b}}{b}\leq \beta \;where\;\beta =\sqrt{\frac{b_{min}}{b_{max}}}$$

If the formula of the estimated plant is defined as exactly the same as the ideal plant, the sliding condition can be defined as shown in [case A] and the control input (u) can be calculated.

[case A] If model is perfect,

$$\dot{s}=\overset{\ldots }{\tilde{x}}+2\lambda \ddot{\tilde{x}}+\lambda^{2}\dot{\tilde{x}}=\dddot{x}-{\dddot{x}}_{d}+2\lambda \ddot{\tilde{x}}+\lambda^{2}\dot{\tilde{x}}=\hat{f}+\hat{b}u-{\dddot{x}}_{d}+2\lambda \ddot{\tilde{x}}+\lambda^{2}\dot{\tilde{x}}$$

$$s\dot{s}=s\left(\hat{f}+\hat{b}u-{\dddot{x}}_{d}+2\lambda \ddot{\tilde{x}}+\lambda^{2}\dot{\tilde{x}}\right)\leq -\eta \left|s\right|$$

$$i)s>0:\;\hat{f}+\hat{b}u-{\dddot{x}}_{d}+2\lambda \ddot{\tilde{x}}+\lambda^{2}\dot{\tilde{x}}\leq -\eta$$

$$u={\hat{b}}^{-1}\left(-\hat{f}+{\dddot{x}}_{d}-2\lambda \ddot{\tilde{x}}-\lambda^{2}\dot{\tilde{x}}-k\right)\;\left(k>\eta \right)$$

$$ii)s<0:\;\hat{f}+\hat{b}u-{\dddot{x}}_{d}+2\lambda \ddot{\tilde{x}}+\lambda^{2}\dot{\tilde{x}}\geq \eta$$

$$u={\hat{b}}^{-1}\left(-\hat{f}+{\dddot{x}}_{d}-2\lambda \ddot{\tilde{x}}-\lambda^{2}\dot{\tilde{x}}+k\right)\;(k>\eta )$$

(24) $$∴u={\hat{b}}^{-1}\left(\hat{u}-ksgn\left(s\right)\right)$$

where

$$\hat{u}=-\hat{f}+{\dddot{x}}_{d}-2\lambda \ddot{\tilde{x}}-\lambda^{2}\dot{\tilde{x}}$$

$$sgn\left(s\right)=\{\begin{array}{cc} +1 & :\;\;s>0\\ -1 & :\;\;s<0 \end{array}.$$

The modeling formula of the estimated plant is uncertain and, if different from that of the ideal plant, can be solved like [case B].

[case B] If model is not perfect,

$$s\dot{s}=s\left(f+bu-{\dddot{x}}_{d}+2\lambda \ddot{\tilde{x}}+\lambda^{2}\dot{\tilde{x}}\right)\leq -\eta \left|s\right|$$

$$=s\left(f+b{\hat{b}}^{-1}\left(\hat{u}-ksgn\left(s\right)\right)-{\dddot{x}}_{d}+2\lambda \ddot{\tilde{x}}+\lambda^{2}\dot{\tilde{x}}\right)$$

$$=\left(f-b{\hat{b}}^{-1}\hat{f}\right)s+\left(1-b{\hat{b}}^{-1}\right)\left(-{\dddot{x}}_{d}+2\lambda \ddot{\tilde{x}}+\lambda^{2}\dot{\tilde{x}}\right)s-b{\hat{b}}^{-1}k\left|s\right|\leq -\eta \left|s\right|.$$

The $b^{-1}\hat{b}$ on both sides of the above equation are multiplied and rearranged as follows:

$$\{b^{-1}\hat{b}f-\hat{f}+\left(b^{-1}\hat{b}-1\right)\left(-{\dddot{x}}_{d}+2\lambda \ddot{\tilde{x}}+\lambda^{2}\dot{\tilde{x}}\right)\}s+b^{-1}\hat{b}\eta \left|s\right|\leq k\left|s\right|$$

$$\left(LHS\right)\leq |b^{-1}\hat{b}f-\hat{f}+\left(b^{-1}\hat{b}-1\right)\left(-{\dddot{x}}_{d}+2\lambda \ddot{\tilde{x}}+\lambda^{2}\dot{\tilde{x}}\right)|\left|s\right|+b^{-1}\hat{b}\eta \left|s\right|\leq k\left|s\right|\left(=RHS\right)$$

$$∴k\geq |b^{-1}\hat{b}f-\hat{f}+\left(b^{-1}\hat{b}-1\right)\left(-{\dddot{x}}_{d}+2\lambda \ddot{\tilde{x}}+\lambda^{2}\dot{\tilde{x}}\right)|+b^{-1}\hat{b}\eta$$

$$\left(RHS\right)=|b^{-1}\hat{b}\left(f-\hat{f}\right)+\left(b^{-1}\hat{b}-1\right)\left(\hat{f}-{\dddot{x}}_{d}+2\lambda \ddot{\tilde{x}}+\lambda^{2}\dot{\tilde{x}}\right)|+b^{-1}\hat{b}\eta$$

$$\leq \beta F+\left|\beta -1\right|\left|\hat{u}\right|+\beta \eta \leq k$$

(25) $$∴k=\beta \left(F+\eta \right)+\left|\beta -1\right|\left|\hat{u}\right|.$$

In this paper, based on the above theory, the plant model Equation (17), inferred from Section 3 in the generalized form as Equation (23), can be expressed as follows:

(26) $$\dddot{x}=-\frac{4\beta_{e}{\bar{A}}^{2}}{mVt}\dot{x}-\frac{\dot{f}\left(x\right)}{m}-\frac{{\dot{F}}_{L}}{m}+\frac{4\beta_{e}\bar{A}{\tilde{s_{r}}}^{-1}D_{m}}{mVt}u$$

where

$$f\left(x,\dot{x},\ddot{x}\right)=-\left(\frac{4\beta_{e}{\bar{A}}^{2}}{mVt}+\frac{K}{m}\right)\dot{x}-\frac{B}{m}\ddot{x}-\frac{{\dot{F}}_{L}}{m}$$

$$b=\frac{4\beta_{e}\bar{A}{\tilde{s_{r}}}^{-1}D_{m}}{mVt},\;{\hat{b}}^{-1}=\frac{\hat{m}Vt}{4\beta_{e}\bar{A}{\tilde{s_{r}}}^{-1}D_{m}}.$$

This process allows the design of sliding mode controllers with modeling uncertainty. The three parameters shown in Equation (27) act as the main factors of the SMC, and the proper adjustment of these values can be seen as the core of nonlinear control. Among other things, the λ and η values contained in the k parameter operate as the main design variables, which speed the response and reduce the tracking error.

(27) $$\{\begin{array}{c} u={\hat{b}}^{-1}\left(\hat{u}-ksat\left(\frac{s}{\Phi }\right)\right)\\ k=\frac{1}{\hat{m}}\left(\Delta K_{max}\dot{x}+\Delta B_{max}\ddot{x}+\Delta {\dot{F_{L}}}_{max}+\hat{m}\left|1-\beta \right|\left|{\dddot{x}}_{d}-2\lambda \ddot{\tilde{x}}-\lambda^{2}\dot{\tilde{x}}\right|\right)+\beta \eta \\ s=\ddot{\tilde{x}}+2\lambda \dot{\tilde{x}}+\lambda^{2}\tilde{x} \end{array}$$

where

$$sat\left(\frac{s}{\Phi }\right)=\{\begin{array}{cc} s/\Phi & :\;if\;\left|s/\Phi \right|\leq 1\\ sgn\left(s/\Phi \right) & :otherwise \end{array}.$$

Another consideration for the SMC is to reduce the chattering of control inputs. Chattering refers to the switching of a variable from a control input around a reference point in a short period of time, which cannot be applied to an input from the actual system, and thus leads to the introduction of the concept of the boundary layer [41]. To apply the boundary layer concept, the existing signum function was replaced with the saturation function.

**Figure 9:**
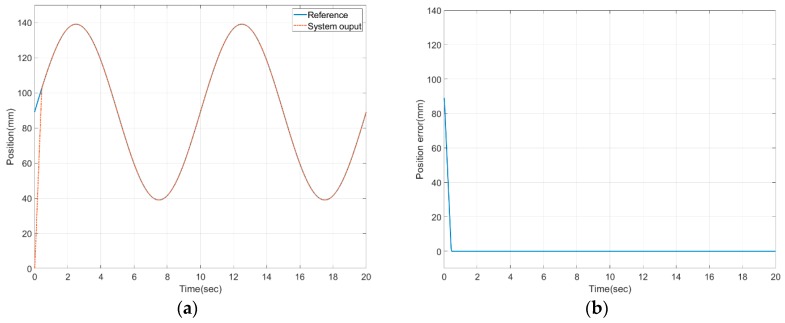
Simulation results of sliding mode control: (**a**) results of position performance in mm; (**b**) tracking error in mm.

Figure 9 demonstrates that the proposed control reached the target value well in Simulink [42]. Since this was a result of an ideal environment, it was possible to see that the output strongly followed the reference input once the error was converged to zero. All the parameter values in the simulation were set according to the values shown in Table 2, based on the actual values of the components that comprised the polycentric knee exoskeleton.

## 5. Experimental Setting and Results

**Figure 10:**
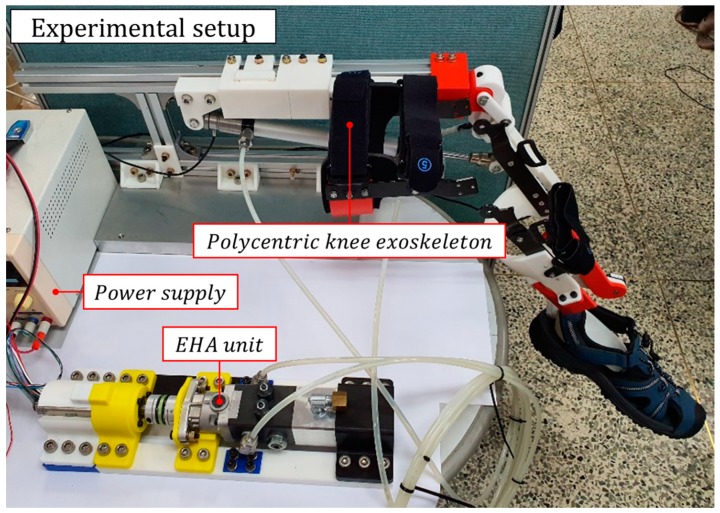
The overall experimental setup attached to a rigid body.

**Figure 11:**
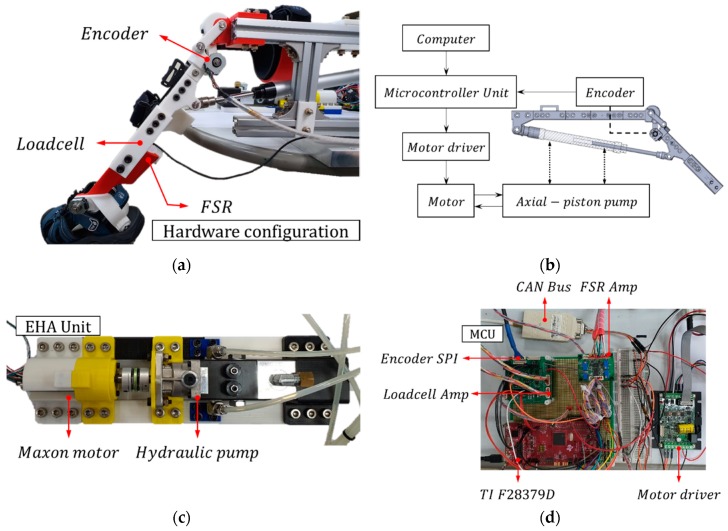
The experimental setup: (**a**) hardware configuration including encoder, loadcell, and force-sensing resistor (FSR); (**b**) schematic of experimental configuration; (**c**) EHA unit with motor and pump; (**d**) microcontroller unit and motor driver for motor control.

An experimental setup was used, as shown in Figure 10 and Figure 11, to validate the proposed controller. The EHA system consisted of a hydraulic cylinder with a maximum permissible pressure of 3.5 MPa, a TFH-080-U-SV (Takako, Seika, Japan) hydraulic pump, and a 200 W Maxon (Maxon motor ag, Sachseln, Switzerland) motor with a gear ratio of 6:1. The motor was controlled by a 0.002 s interrupt on a Texas Instruments F28379D system clock at 200 MHz and transmitted and received data via controller area network (CAN) communication with the motor driver. Lastly, the AMT203-V rotary encoder (CUI Devices, Tualatin, OR, USA), which was used as a feedback sensor, transmitted data to the microcontroller unit (MCU) via serial peripheral interface (SPI) communication. The detailed hydraulic parameters for the EHA experiment are shown in Table 3.

**Figure 12:**
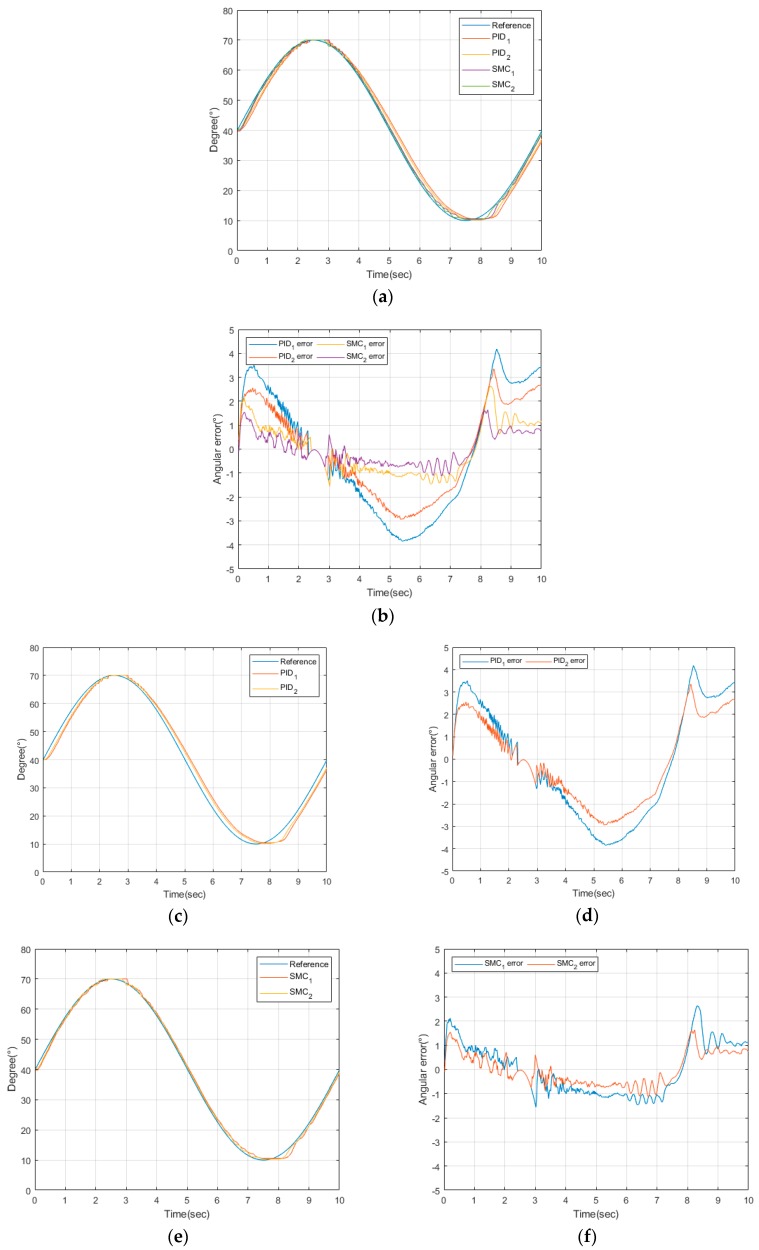
Experimental results of sliding mode control and proportional-integral-differential (PID) control: (**a**,**c**,**e**) results of angular position performance; (**b**,**d**,**f**) angle tracking error.

As shown in Figure 12, it was found that the polycentric knee exoskeleton robot followed the target values well when it was given a sinusoidal input angle. For the experimental environment, the range of the corresponding cylinder length values of 38.98 mm to 139.05 mm was set when the knee angle range was 10° to 70°. In other words, a system was implemented in which the cylinder rod operated by about 200.14 mm for a 0.1 Hz sine wave period. Later, we performed gain tuning to improve the system’s following response to the target values by making changes to the design parameter values, and we also analyzed the effects of each parameter on the system. In addition, after the optimal tuning of proportional-integral-differential (PID) controllers, which are commonly used in the field, we found that the sliding mode controller, which is shown in Figure 12b, had better performance. While the angle following error was less than 2° when the SMC was applied, there was a gap that varied up to approximately 4° when PID was applied.

The initial error size, as seen in Figure 12d,f, was somewhat greater than in other intervals because the difference was larger between the current value and the reference input value during the time that the motor was accelerating while stationary. However, once the sliding surface was reached, continuous follow-up could be seen. The reason why the error did not converge to zero, unlike the ideal system, was that the Maxon motor’s permissible maximum speed was limited to 5000 rpm to ensure the stability of the motor. It was also thought that the effects of external disturbances may have affected the error.

**Figure 13:**
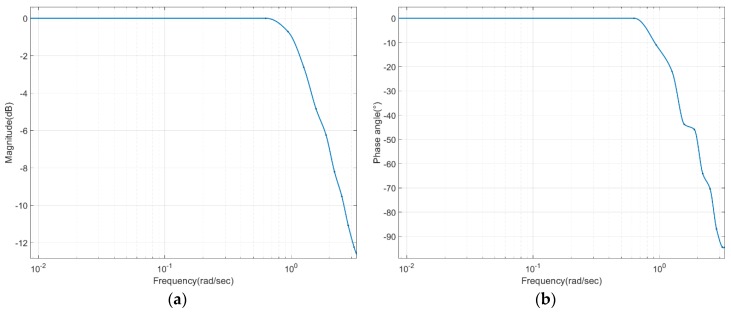
Driving capacity as frequency changes: (**a**) magnitude variation in angle tracking; (**b**) phase variation in angle tracking.

Further experiments were then conducted by altering the frequency of the reference input to check the driving capacity for angular tracking. The experiment was conducted five times for each frequency and received responses for sine inputs of more than three cycles. By fitting these data into the shape-preserving (PCHIP) method, we obtained magnitude and phase plots, as shown in Figure 13. From the graph, we could see that the frequency at the −3 dB point was about 0.21 Hz, which gave us a bandwidth for angular tracking.

## 6. Discussion

In this paper, a simple 1 DoF system, which has mainly been used in the knee structures of exoskeleton robots, was designed as a polycentric structure with minimal misalignment between wearer and robot, and the robot’s knee angle was controlled using an energy-efficient EHA system. A sliding mode control with a feedback sensor was applied to solve the problems of mechanical uncertainty arising from this process.

We were able to verify that the behavior of the robot, which included the EHA system, was different due to changes in the values of several design parameters. In general, the larger the value of λ, the faster the robot attempted to follow the desired target value, but the change in the value of λ affected the sliding condition s(t), so it was important to select a proper Φ value to control it. It was confirmed that failure to do so resulted in chattering. It was also found that the k value had a significant effect on the sliding mode control. Among the variables, it was found that η was the variable that most directly affected k, and the k value was also increased as the η value became larger, making the system more adherent to the target value. However, we found it important to determine the appropriate k value through experimental methods, since indiscriminately making the k value larger introduces instability in the system or puts it in a situation where it cannot be driven due to hardware limitations.

For verification of the SMC designed, we compared the performance of the controller by configuring the most commonly used PID controller for general control. The experimental results confirmed that SMCs are more efficient in solving the uncertainty of plant models and the nonlinearity of EHAs. Experimental data may question performance as there is no significant difference in values. However, if the system is applied to a real person, it will include more mechanical uncertainty, and the SMC can be expected to achieve better performance because of the presence of variables to address these mechanical uncertainties. Although it can be seen that some errors exist for reference input, these can be resolved by improving the specifications of the motor used in the system configuration or by increasing the resolution of the feedback sensor, interpreting information about external disturbance, and selecting appropriate control parameters. 

In order to measure the driving capacity of a robot, we experimented with changing the frequency of the reference input to determine the bandwidth of the designed robot’s angular tracking. The frequency response confirms that the value corresponding to the −3 dB point is approximately 0.21 Hz. This is slower than normal people’s walking speed, but it is believed to be suitable for passive rehabilitation training. Since the performance problem is caused by limiting the allowable maximum speed of the motor, as described above, and the flow rate of the pump is somewhat low, we think it is a system that is sufficiently feasible with a real exoskeleton if the specifications of the motor and the pump are improved.

## 7. Conclusions and Future Work

Various attempts are being made to develop wearable robots, including the development of mechanisms for rehabilitation exoskeleton robots, the systemization of training methods, and the upgrading of control methods. The present study proposed a structure that minimizes misalignment through ergonomic designs of knee structures, which were overlooked by previous studies, to transfer power to wearers without loss of energy. In addition, sliding mode controllers were designed to control this complex structure with energy-efficient EHAs, and to solve the problems of mechanical uncertainty and nonlinearity of fluids that arise in the process. Although the driving capacity was not high because the −3 dB frequency was 0.21 Hz in this study, by configuring the system with limited performance products, a series of experiments showed meaningful results. Therefore, designing and controlling polycentric knee exoskeleton robots using the EHA system is thought to have great potential and is considered to be a foundation for the development of wearable robots.

For future work, it will be necessary to ensure hardware stability by increasing the specifications used in the system configuration along with more accurate mechanical modeling of exoskeleton robots. This will be a fundamental solution to increasing the driving capacity of the robot proposed in this study. For example, improvement of the speed control capability of a motor by upgrading its rpm specifications or acceleration/deceleration performance, improving the flow of the pump, and improving the resolution of the feedback sensor should be performed. In addition, the system should be optimized to enable the robot to provide direct assistance when walking, in accordance with the wearer’s intentions, with studies such as torque control and disturbance analysis.

## Figures and Tables

**Table 1:** Coefficient of $\theta_{p}$ and x polynomial.

P-Value		Q-Value	
$p_{1}$	1.571 × 10^−8^	$q_{1}$	–1.807 × 10^−9^
$p_{2}$	–4.164 × 10^−6^	$q_{2}$	5.788 × 10^−7^
$p_{3}$	0.0004789	$q_{3}$	–7.285 × 10^−5^
$p_{4}$	–0.03192	$q_{4}$	0.004161
$p_{5}$	–0.6195	$q_{5}$	–0.6389
$p_{6}$	148	$q_{6}$	91.73

**Table 2:** Parameters for electro-hydraulic actuator (EHA) simulation.

Parameter	Specification
x	≤150 mm
u	≤5000 rpm
$V_{t}$	$41,233\;{\mathrm{mm}}^{3}$
m	1.6 kg (1.4 kg ≤ m ≤ 1.8 kg) $\hat{m}=\sqrt{2.52}≅1.587\;\mathrm{kg}$
$\beta_{e}$	17,200 bar
$\bar{A}$	$274.8894\;{\mathrm{mm}}^{2}$
B	0.05 N/(m/s)
$D_{m}$	0.8 cc/rev

**Table 3:** Detailed hydraulic parameters for the EHA experiment.

Component	Parameter	Specification	Component	Parameter	Specification
Hydraulic cylinder	Bore size	20 mm	Motor	Input voltage	24 V
	Rod size	10 mm		Watts	200 W
	Maximum allowable pressure	3.5 MPa		Speed limit	5000 rpm
	Stroke length	150 mm	MCU	System clock	200 MHz
Hydraulic pump	Displacement	0.8 cc		Interrupt time	0.002 s
Hydraulic oil	Model	ISO VG 46	Mass	Weight	1.6 kg (shank)
	Bulk modulus	17,200 bar	Encoder	Degree	0–360°

## Abbreviations

The following abbreviations are used in this manuscript:

DoF	Degree of freedom
EHA	Electro-hydraulic actuator
SMC	Sliding mode controller
ACL	Anterior cruciate ligament
PCL	Posterior cruciate ligament
ICR	Instantaneous center of rotation
SEA	Series elastic actuator
HA	Hydraulic actuator
ROM	Range of motion
MCU	Microcontroller unit
SSE	Sum of square error
RMSE	Root mean square error
BLDC	Brushless DC
I/O	Input/output
CAN	Controller area network
SPI	Serial peripheral interface
FSR	Force-sensing resistor
PID	Proportional-integral-differential
PCHIP	Piecewise cubic Hermite interpolation
**Nomenclature**	
$\theta_{p}$	Relative angle between the ground link and the point P within the coupler (°)
x	Position (mm)
$p_{n}$	Coefficients of polynomial with respect to $\theta_{p}$ (−)
$q_{n}$	Coefficients of polynomial with respect to x (−)
$D_{m}$	Ideal volumetric displacement of the motor $\left({\mathrm{mm}}^{3}/\mathrm{rev}\right)$
${\dot{\theta }}_{m}$	Motor shaft speed (rev/s)
$Q_{1}$	Return flow from motor in pump, supplied flow in cylinder $\left({\mathrm{mm}}^{3}/s\right)$
$Q_{2}$	Forward flow to motor in pump, return flow in cylinder $\left({\mathrm{mm}}^{3}/s\right)$
$C_{im}$	Internal or cross-port leakage coefficient $\left({\mathrm{mm}}^{3}/s/\mathrm{bar}\right)$
$C_{em}$	External leakage coefficient $\left({\mathrm{mm}}^{3}/s/\mathrm{bar}\right)$
$P_{1}$	Pressure in the return chamber (bar)
$P_{2}$	Pressure in the forward chamber (bar)
$Q_{L}$	Load flow $\left({\mathrm{mm}}^{3}/s\right)$
$P_{L}$	Pressure difference (bar)
$V_{1}^{0},\;V_{2}^{0}$	Two chambers of initial condition $\left({\mathrm{mm}}^{3}\right)$
$\beta_{e}$	Bulk modulus (bar)
$P_{r}$	Reference pressure (bar)
$C_{{im}^{\prime }}$	Coefficient of the internal leakage $\left({\mathrm{mm}}^{3}/s/\mathrm{bar}\right)$
$C_{em1^{\prime }}$	Coefficient of the external leakage from the return chamber $\left({\mathrm{mm}}^{3}/s/\mathrm{bar}\right)$
$C_{{em2}^{\prime }}$	Coefficient of the external leakage from the forward chamber $\left({\mathrm{mm}}^{3}/s/\mathrm{bar}\right)$
$A_{1},\;A_{2}$	Area of each chamber $\left({\mathrm{mm}}^{2}\right)$
$V_{t}$	Total hydraulic actuator volume $\left({\mathrm{mm}}^{3}\right)$
$\bar{A}$	Average of cross-sectional area of chamber $\left({\mathrm{mm}}^{2}\right)$
m	Mass of the load (kg)
B	Damping coefficient (N/(m/s))
K	Spring coefficient (N/m)
$F_{L}$	Disturbance (N)
u	Motor shaft speed, control input (rev/min)
$\tilde{s_{r}}$	Scaling factor (−)
s	Sliding surface (−)
λ	Strictly positive constant (−)
$x_{d}$	Desired position (mm)
e	Tracking error (mm)
η	Design parameter (−)
Φ	Boundary layer thickness (−)
